# Snow water scarcity induced by record-breaking warm winter in 2020 in Japan

**DOI:** 10.1038/s41598-020-75440-8

**Published:** 2020-10-29

**Authors:** Satoshi Watanabe, Shunji Kotsuki, Shinjiro Kanae, Kenji Tanaka, Atsushi Higuchi

**Affiliations:** 1 School of Engineering, The University of Tokyo, Tokyo, 113-8656 Japan; 2 Center for Environmental Remote Sensing (CEReS), Chiba University, Chiba, 263-8522 Japan; 3 RPRESTO, Japan Science and Technology Agency, Chiba, Japan; 4 RIKEN Center for Computational Science, Kobe, Japan; 5 School of Environment and Society, Tokyo Institute of Technology, Tokyo, 152-8550 Japan; 6 Disaster Prevention Research Institute, Kyoto University, Uji, Kyoto 611-0011 Japan

**Keywords:** Climate sciences, Hydrology

## Abstract

This study highlights the severity of the low snow water equivalent (SWE) and remarkably high temperatures in 2020 in Japan, where reductions in SWE have significant impacts on society due to its importance for water resources. A continuous 60-year land surface simulation forced by reanalysis data revealed that the low SWE in many river basins in the southern snowy region of mainland Japan are the most severe on record. The impact of the remarkably high temperatures in 2020 on the low SWE was investigated by considering the relationships among SWE, temperature, and precipitation. The main difference between the 2020 case and prior periods of low SWE is the record-breaking high temperatures. Despite the fact that SWE was the lowest in 2020, precipitation was much higher than that in 2019, which was one of the lowest SWE on record pre-2020. The results indicate the possibility that even more serious low-SWE periods will be caused if lower precipitation and higher temperatures occur simultaneously.

## Introduction

Japan experienced lowest snowfall in many regions in the boreal winter 2020. Lowest snowfall results in the lowest amount of snow water equivalent (SWE). Reductions in SWE have significant impacts on society, especially in Japan. Cities in northern Japan are the snowiest cities in the world, receiving more than 600 cm of snowfall annually. Snow water plays an important role in terms of water resources in Japan, and many efforts have been devoted for the estimation^1^.

Increasing temperatures worldwide are leading to increased interest in snow levels^2^. This holds true for Japan as well, since many cities, including Tokyo, the capital of Japan, rely on snow water for some proportion of water resources. In fact, water usage restrictions due to low SWE were imposed in 2019. Since low SWE levels also occurred in 2020, i.e. 2 years in a row, there is increasing need for accurate understanding of SWE under global warming conditions. The boreal winter of 2020 was among the warmest on record, which was also the case in Japan.

Many studies have been conducted to understand and project the impacts of global warming. Prior to the recent period of low SWE in Japan, a significant reduction due to global warming was already projected^3,4^. Especially, remarkable reduction in snow cover and snowfall are projected in years with light snow cover^5^. It would be informative to investigate the low SWE period in Japan in 2020, characterised by low SWE with remarkably high temperatures, as it represents a case under conditions of global warming, something that previous studies have tried to project.

This study analyses the case of remarkably low SWE in Japan in 2020 by comparing it with past cases of low SWE. Given the aforementioned impacts of low SWE, the analysis of the record-breaking case of 2020 is expected to be beneficial for future predictions and policy-making. The aim of this study was to compare the 2020 case with past cases, understand its degree of severity, and identify similarities and differences with past cases. Because of the significant North–South span of Japan, snow tendencies are diverse within the country. Consideration of this diversity is necessary for the analysis.

We herein performed a continuous 60-year land surface simulation with the Simple Biosphere Model including Urban Canopy model (SiBUC;^6,7^). The Japanese 55-year Reanalysis (JRA55;^8^) was used as forcing data to drive the model (see Methods “Land surface simulations” for details). We performed preliminary SiBUC-based simulations at seven observational sites to validate the snow module, and confirmed the ability to reproduce past SWE levels (Supplementary Figure S2). Kawase et al.^9^ used JRA55 as forcing data and successfully simulated the daily variations in snow depth in the Japanese Northern Alps. The SiBUC computes the SWE, which enables comparisons over long time periods and wide areas. Another possible approach to comparing the 2020 case with past examples would be to use observed measurements. However, as the locations and time frames for which observations are available are limited, such measurements are unsuitable for wide-ranging, long-term comparisons. The use of a method employing satellite observations could also be considered; in recent years, a wealth of satellite information has become available, yielding a trove of research results. This method is suitable for comparisons over a wide area, but cannot provide information for past time periods when satellite observations were not available. In addition, some challenges remain in the estimation of regional snow trends^10^.

In this study the severity of low snowfall levels in 2020 was quantified from the SWE variability obtained from a continuous 60-year land surface simulation. The SWE variability explained by temperature and precipitation was also quantified to understand the meteorological background on low SWE. To conduct a long-term comparison spanning a wide area, an index capable of standardising characteristics that differ for each region is necessary. An index that indicates severity in terms of the number of standard deviations from the long-term mean, was used to quantify the severity of low snowfall levels, which allows severity to be assessed and compared between multiple regions with differing average values (see Methods “Severity index” for details).

## Results

**Figure 1:**
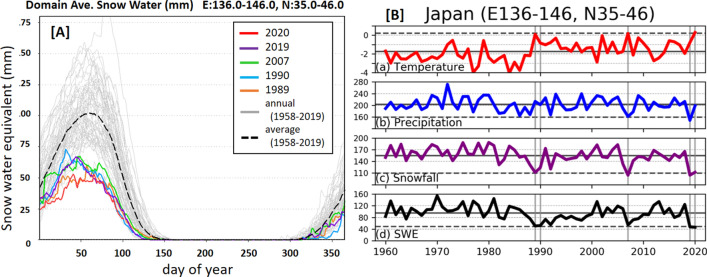
(**A**) Seasonal evolution in domain-averaged SWE and (**B**) 60-year change in the domain-averaged temperature, precipitation, snowfall, and SWE. In (**A**), seasonal evolution from 1961 to 2020 are indicated by a 60-year average (dashed line), and the years with lowest five SWE are indicated in colours: 1989(orange); 1990 (blue); 2007 (green); 2019 (purple); and 2020 (red). The horizontal axis represents day of year. In (**B**), the temperature is averaged over January to February, precipitation and snowfall are shown as the totals during January and February, and the SWE is averaged over the period from the 1st to the 10th of March, as decided based on the seasonal evolution results. Vertical lines delineate five lowest SWE years. The bold and dashed horizontal line delineates mean of 60 years and the value equivalent to two sigma of standardised index calculated for domain average.

Figure 1 shows the seasonal evolution and long-term changes in SWE in mainland Japan obtained by the 60-year continuous land surface simulation. The SWE is spatially averaged over the domain 136.0–146.0 E and 35.0–46.0 N, which includes most of the snowy area of mainland Japan. The results show that the peak of the SWE occurred from late February to early March. Thus, a 60-year time series was calculated by averaging SWE from the 1st to the 10th of March. The calculation from the 1st to the 10th of April was also examined, and confirmed that our main conclusions were robust to the change of period (Supplementary Figure S1). In addition to the SWE, the 60-year time series of mean temperature, total precipitation, and total snowfall from January to February were also examined because high temperatures and low precipitation are considered to be two possible reasons for the low SWE.

The results show that the SWE in Japan in 2020 was one of the lowest, equivalent to the levels in 1989, 1990, 2007, and 2019. According to the figure indicating seasonal evolution (Fig. 1A), the SWE in 2020 was significantly lower than the 60-year average, and lower than that of most other years for almost every day of the year.

According to the time series shown in Fig. 1B, the mean temperature from January to February 2020 was highest in the record. The total precipitation from January to February in 2020 was near the average level. The total snowfall from January to February in 2020 is close to the lowest in the record. In other words, the low snowfall was not due to low precipitation but rather due to high temperatures, which leads to a situation characterised by more precipitation occurring as rainfall than as snowfall. The situation in 2020, characterised by low SWE, high temperatures, and average precipitation, is the same as the situation in 1989 and 1990. On the other hand, the case of 2019, the other low SWE years, is characterised by precipitation lower than average. The case of 2007 is characterised by both high temperature and low precipitation while the temperature is lower than in 2020 and precipitation is higher than in 2019. To understand the relationship among them, it is necessary to examine the analysis at a specific spatial scale.

**Figure 2:**
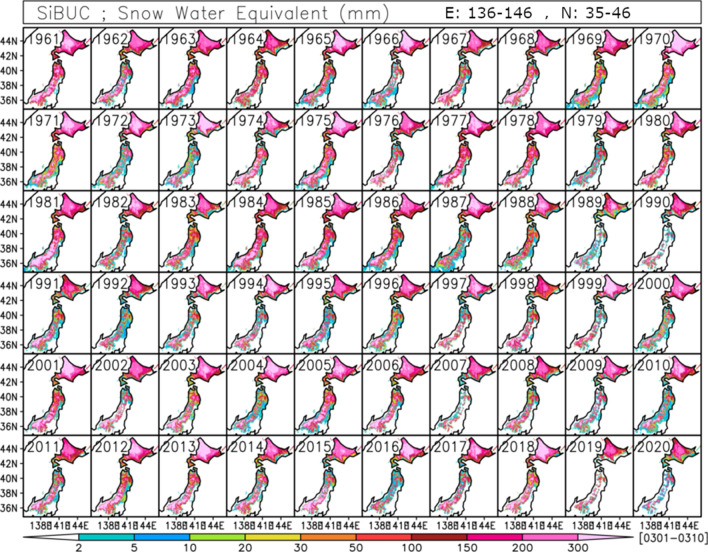
Spatial distribution of SWE averaged over the period from March 1st to March 10th from 1961 to 2020.

The SWE in 2020 is one of the lowest across mainland Japan, except for in the regions from 39.0 to 41.0 N, which is the northern snowy region of mainland Japan (hereafter referred to as NM). Figure 2 shows the spatial distribution of SWE in Japan over 60 years from 1961 to 2020. The result shows that SWE in 2020 in the domain under the 39.0 N, which is the southern snowy region of mainland Japan (hereafter referred to as SM), is lower than or equivalent to 1989, 1990, 2007 and 2019, which domain-averaged SWE is fairly lower than average. This is significant especially in the southern regions.

In order to understand the characteristics of the SWE at a specific spatial scale and its impact on water resources, we investigated the average SWE over river basins meeting the criterion that the area is more than 1000 km^2^ and the basin average SWE is greater than 5 mm for a 60-year average. Here, the standardised index was used to quantify the severity during the target period and to compare the severity between the river basins. In addition, a multiple regression analysis was conducted to quantify the SWE variability explained by temperature and precipitation.

**Figure 3:**
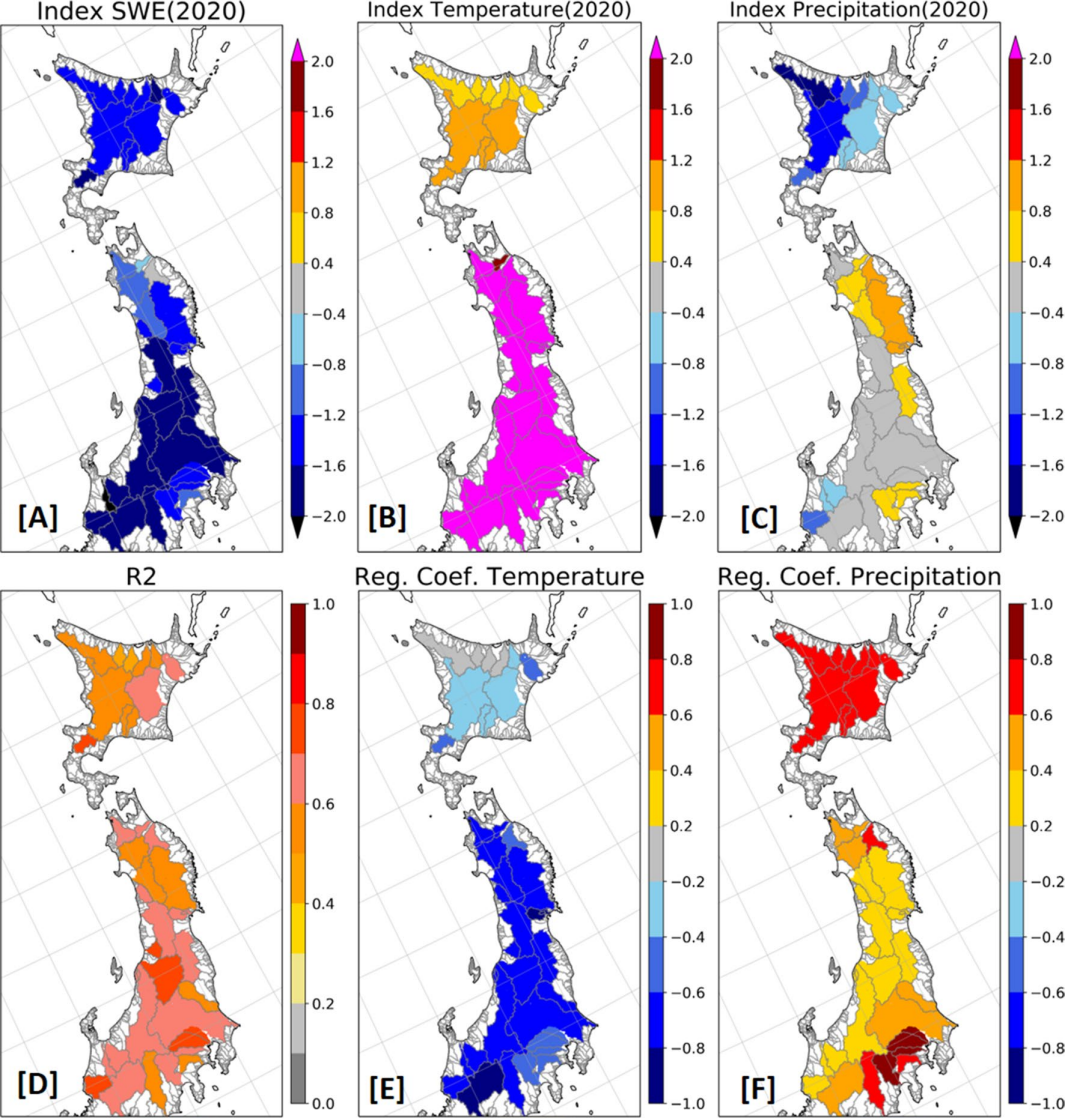
Standardised indices for average SWE (**A**), average temperature (**B**), and total precipitation (**C**) in 2020 and the coefficient of determination (**D**) and the standardised partial regression coefficient of temperature (**E**) and precipitation (**F**) from the result of multiple regression analysis in each major basin.

Figure 3 shows the calculated standardised index of SWE, temperature, and precipitation, which indicates the SWE indices in most river basins are lower than − 1.2, which corresponds to that exceedance probability is approximately 11.5%. In the southern target river basins, they are lower than − 1.6 and 5.5%. The SWE in 2020 is record-breaking in 8 of 36 river basins. In addition, the temperature indices in most river basins are more than 2.0, corresponding to that exceedance probability is approximately 2.5%, and are record-breaking in 21 of 36 basins. While the precipitation indices in some of northern river basin is lower, they are not significant in most river basins.

In 2020, the standardised index of temperature in river basins of the SM are higher and that of precipitation is lower in river basins of the domain over 41.0 N including Hokkaido, the north island of Japan, which results in the lower SWE in both basins of the SM and Hokkaido. The result of a multiple regression analysis shows that SWE variability is explained by temperature more than precipitation in the basins of the SM, and vice versa in Hokkaido. The coefficient of determination of a multiple regression analysis indicates almost half of the SWE variability is explained by the variability of temperature and precipitation.

**Figure 4:**
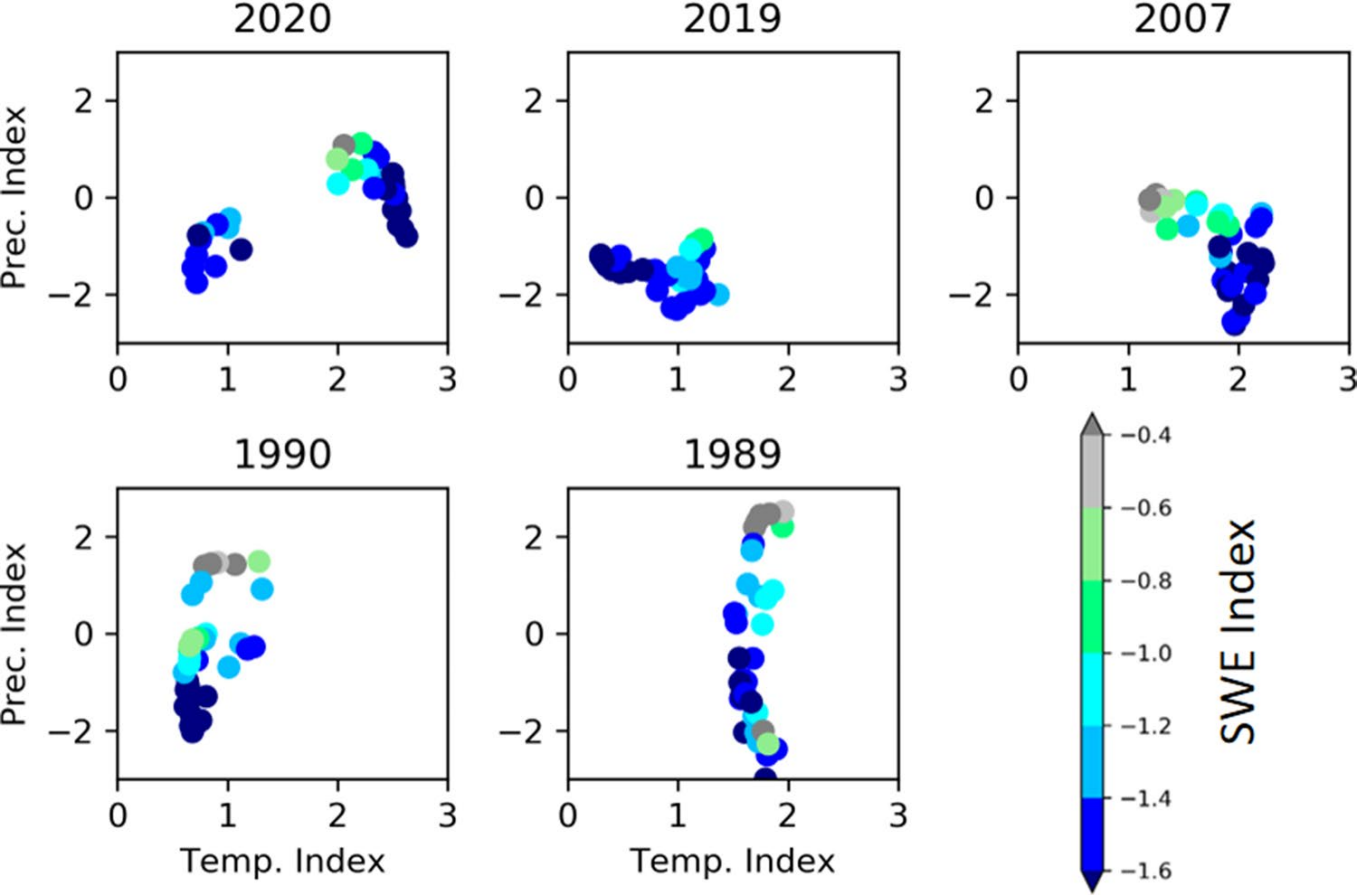
Relationship among standardised indices in major basins for the five lowest-SWE years. The estimated standardised index of SWE is indicated by the colour gradient, and plotted along with temperature (horizontal axis) and precipitation (vertical axis). Data points in the bottom-right quadrant represent basins characterised by higher temperatures and lower precipitation than the respective averages.

The characteristic of the SWE in 2020, which is the combination of higher temperature in basins of the SM and lower precipitation in basins of Hokkaido, is unique comparing to other lower SWE years. Figure 4 shows the relationship among standardised indices in major basins for the five lowest-SWE years. The relationship is different in each of the lowest 5 years. Each lowest year is explained by low precipitation and/or high temperature. In 2007, explained by both higher temperature and lower precipitation, SWE is not lower than in 2020 because temperature is lower than in 2020. In 2020, the river basins characterized by high temperature have higher temperature than other years, and the river basins characterized by low precipitation are comparable to other years. The results indicate the possibility that lower SWE than in 2020 will be caused if temperature in 2020 and precipitation in 2007 or 2019 occur simultaneously.

## Discussion

The results indicated that the relationships between the severity of SWE, temperature, and precipitation differ by region. In the basins of the SM, temperature is more important than precipitation for the SWE. This is in line with the findings of previous studies^11–13^. Warmer temperatures lead to the conversion of snowfall to rainfall^14^. Since the mean temperature in the basins of the SM is higher than those in the basins of the NM and Hokkaido, the influence of temperature is relatively greater in the basins of the SM.

The record-breaking low SWE in 2020 along with the highest temperatures seems to validate the results of previous studies assessing the impact of global warming on SWE. Some studies (e.g.^3,4^) pointed to the significant impact of increased temperatures on reductions in SWE based on future projections using climate simulations. As shown in the results, the temperature in 2020 was significantly higher than the average except for the basins of Hokkaido. The temperature in 2020 was more than 2° above the average. The situation in 2020 is indicative of a situation under climate change. The projections in above mentioned studies were realized in 2020.

The results also indicate the possibility that precipitation levels lower than those in 2020 would lead to an even lower SWE. As shown in the results, precipitation in the basins of the SM in 2020 was not lower than that in 2019, which is the year with the next-lowest SWE. If the record high temperatures of 2020 and the low precipitation levels of 2019 overlapped, it could lead to the lowest SWE among all results. Kawase et al.^15^ projected the reductions in winter precipitation under global warming on the coast of the Sea of Japan and over the Pacific Ocean in the south of the Japanese archipelago.

Although not record-breaking, the SWEs in the basins of Hokkaido in 2020 were also lower than average, and the situations in the basins of Hokkaido are different from that in the basins of the SM. In the basins of Hokkaido, the temperature is not highest but the precipitation is lower in 2020. In the basins of Hokkaido, average temperatures were relatively low compared to those in the basins of the SM, so the SWE in the basins of Hokkaido may be more sensitive to changes in precipitation than to changes in air temperature.

In order to consider the impact of low SWE on water resources, it is necessary to consider the effects of dams and projections of water demand. As mentioned previously, water usage was restricted in Japan in 2019. Although the SWE in 2020 was less than that in 2019, the lower SWE in 2020 would not necessarily lead to decreased water resources because the precipitation in 2020 was greater than that in 2019. It should be noted that the impacts of low SWE and low precipitation on water resources would not be equivalent, due to the difference between precipitation occurring as snowfall or rainfall. The impact of the conversion of snowfall to rainfall on water resources would depend on dams and water demand. Hydrological simulations that consider the difference between snowfall and rainfall, as well as the effects of dams and water demand, will be important for further investigations.

## Methods

### Land surface simulations

Land surface simulations were performed using the Simple Biosphere including Urban Canopy model (SiBUC;^7^), in which water, energy, and radiation budgets on the Earth’s surface are solved. To drive the model, seven meteorological forcing variables are needed: precipitation; short-wave radiation; long-wave radiation; atmospheric pressure; specific humidity; atmospheric temperature; and wind speed. Kotsuki et al.^6^ developed a quasi-real-time system to operate SiBUC-based simulations over Japan. Using this system, we conducted a continuous simulation from January 1958 to March 2020 with 0.04° horizontal resolution using Japanese 55-year Reanalysis (JRA55) forcing data^8^. The horizontal resolution of JRA55 was 1.25°, and was bilinearly interpolated onto the 0.04° considering the topography with 0.04° horizontal resolution following Kotsuki et al.^6^.

The snow module in SiBUC follows Sellers et al.^16^ and was validated by Tanaka et al.^17^. In the snow module, the canopy-intercepted snow and ground snow are treated separately. The sum of canopy-intercepted snow and ground snow is defined as the SWE in this study. The performance of the SiBUC was compared in the Snow Model Intercomparison Project for forest snow processes (SnowMIP2)^18^. We performed preliminary SiBUC-based simulations at seven observational sites to validate the snow module. At the observational sites, snow depth was measured in addition to the meteorological forcing data. With observational (i.e. reliable) forcing data, the model appropriately reproduced seasonal evolution in snow water depth (Supplementary Information; Figure S2 and S3). The results show that the SiBUC simulations forced by JRA55 have the ability to reproduce past SWE levels.

### Severity index

To conduct a long-term comparison over a wide area, an index capable of standardising characteristics that differ by region is necessary. Therefore, this study introduced a standardised index based on the idea of the standardised precipitation index (SPI)^19^, a representative index measuring meteorological drought. SPI represents the probability distribution of precipitation normalised to a normal distribution. The value of the SPI illustrates the frequency based on the sigma value of the distribution. While gamma or other distributions have been used in the SPI, we applied the Box–Cox transformation^20^, which is parametric power transformation into a Gaussian distribution, instead of fitting a probabilistic distribution. We performed a Box–Cox transformation to the mean temperature, total precipitation, and total snowfall from January to February and mean SWE from 1st to 10th March for 1961–2020, which the definition is identical to that used in Fig. 1. The transformation was conducted for each river basin. Thus, the target variables, mean temperature, total precipitation, total snowfall, and SWE were averaged over each river basin before the transformation. The parameter of the Box–Cox transformation, which determines the accuracy of transformation, was decided by maximum likelihood estimation for each variable and river basin separately. The derived standard deviation from transformed variables is the index representing the severity in this study. In this index, the probabilities that the absolute value of the index exceeds 1, 2, or 3 are approximately 16%, 2.5%, and 0.15%, respectively. For example, when evaluating an area where SWE is low, larger negative values imply less-common weather phenomena. Although three month or longer is appropriate for SPI measuring meteorological drought, the index in this study uses ten days for SWE and two months for the other variables because these time period can capture the variability.

## Supplementary information

Supplementary Information
