# Supplementary material for: Snow water scarcity induced by record-breaking warm winter in 2020 in Japan
Source: Sci Rep. 2020 Oct 29;10:18541. doi: 10.1038/s41598-020-75440-8 (PMC7596238; doi:10.1038/s41598-020-75440-8)
Supplement: Supplementary file 1 — Supplementary Information [file 41598_2020_75440_MOESM1_ESM.docx]

**Supplementary Information**

**Snow water scarcity induced by record-breaking warm winter in 2020 in Japan**

**Satoshi Watanabe^1*^, Shunji Kotsuki^2,3,4^, Shinjiro Kanae^5^, Kenji Tanaka^6^, and Atsushi Higuchi^2^**

^1^ School of Engineering, the University of Tokyo, Tokyo, 113-8656, Japan

^2^ Center for Environmental Remote Sensing (CEReS), Chiba University, Chiba, 263-8522, Japan

^3^RPRESTO, Japan Science and Technology Agency, Chiba, Japan

^4^RIKEN interdisciplinary Theoretical and Mathematical Sciences Program, Kobe, Japan

^5^ School of Environment and Society, Tokyo Institute of Technology, Tokyo, 152-8550, Japan

^6^ Disaster Prevention Research Institute, Kyoto University, Uji, Kyoto, 611-0011, Japan

*stswata@hydra.t.u-tokyo.ac.jp

**A 60-year time series calculated from the 1st to the 10th of April instead of March, and their impact to conclusions**

A 60-year change in the domain-averaged SWE from the 1^st^ to the 10^th^ of April was calculated and compared with that of March. This comparison was conducted to understand the impact of the difference of target period on conclusions. The result shows that most of lower SWE years are identical between them although there are some differences especially for the higher SWE years. Three of five lowest SWE years are identical and the other two years are also within tenth lowest. In the present study, this difference was deemed to small impact on the main conclusions because the highest temperature in 2020 is unique even in the lowest SWE years of April.

**Validation of land surface simulations**

Land surface simulations using the Simple Biosphere including Urban Canopy (SiBUC) forced by Japanese 55-year Reanalysis (JRA55) were validated with in-situ observation data known as the Automated Meteorological Data Acquisition System (AMeDAS) from 7 observation stations around the snowy areas of Japan. Seasonal evolution of snow depth (in cm) and mean snow depth from 1st to 10th March were compared for 1990-2019.

The results show that the simulations forced by JRA55 have the ability to reproduce past SWE levels. While there are discrepancies between the model-estimated and observed data around the peak of snow depth in seasonal evolution, increases and decreases in the values were well-reproduced. The coefficient of determination in each observation point shows that interannual variability of mean snow depth from 1st to 10th March were also well-reproduced. In the present study, this gap in consistency was deemed to have a small impact on the main results because the use of the standardised index compensates for the gap sufficiently.

**Supplementary Figures**

**
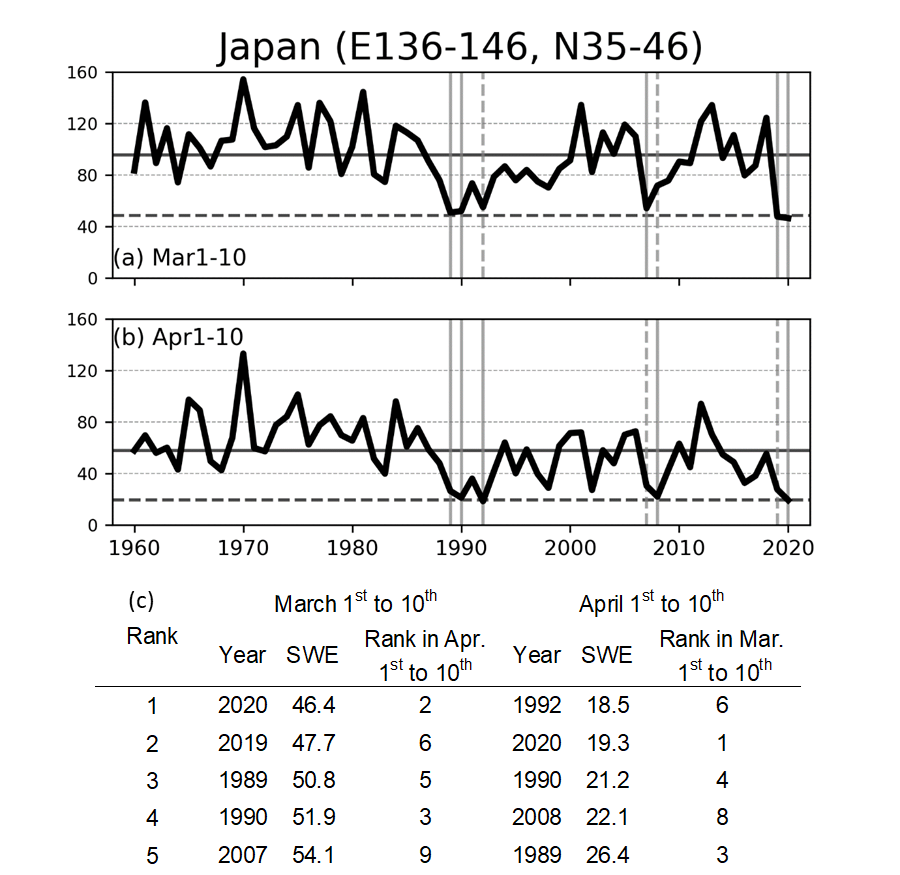
**

**Supplementary Figure S1.** 60-year change in the domain-averaged SWE averaged from the 1^st^ to the 10^th^ of March (a) and April (b), and the table listed five lowest SWE years in each month. In figure (a) and (b), the five lowest SWE years in each month are delineated with vertical bold lines. If the year is one of the five lowest in March and not in April or vice versa, the year is delineated with dashed vertical lines. The horizontal bold and dashed line in figure (a) and (b) show the SWE corresponds to average over 60 years and that standardised index is two.


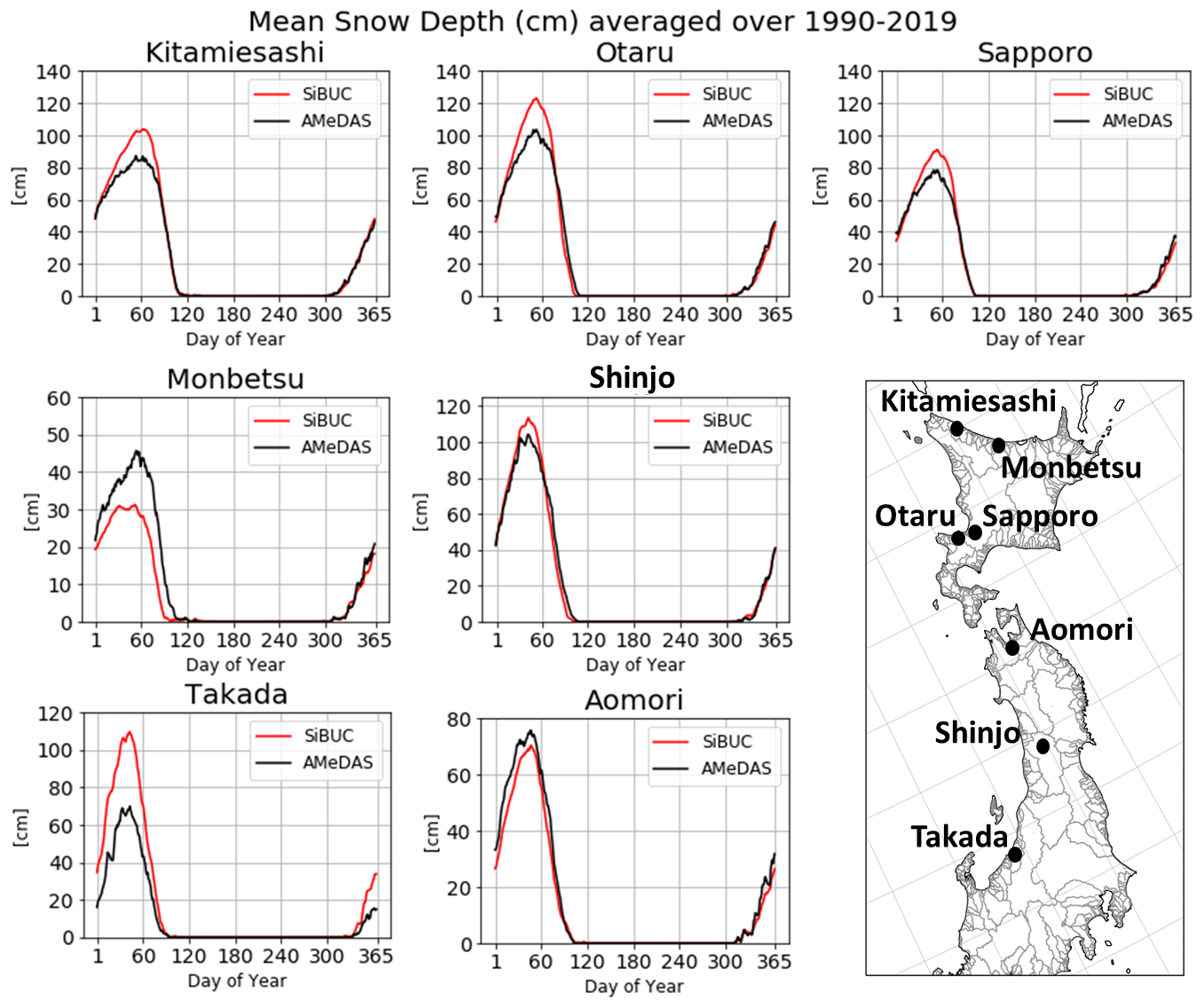


**Supplementary Figure S2.** Seasonal evolution of snow depth (cm) averaged over the period of 1990–2019 determined by SiBUC-based simulations (red) and experimental observation (black) at nine observational sites in Japan. Snow depth was computed from ground snow assuming a snow density of 0.20 g cm^-3^.


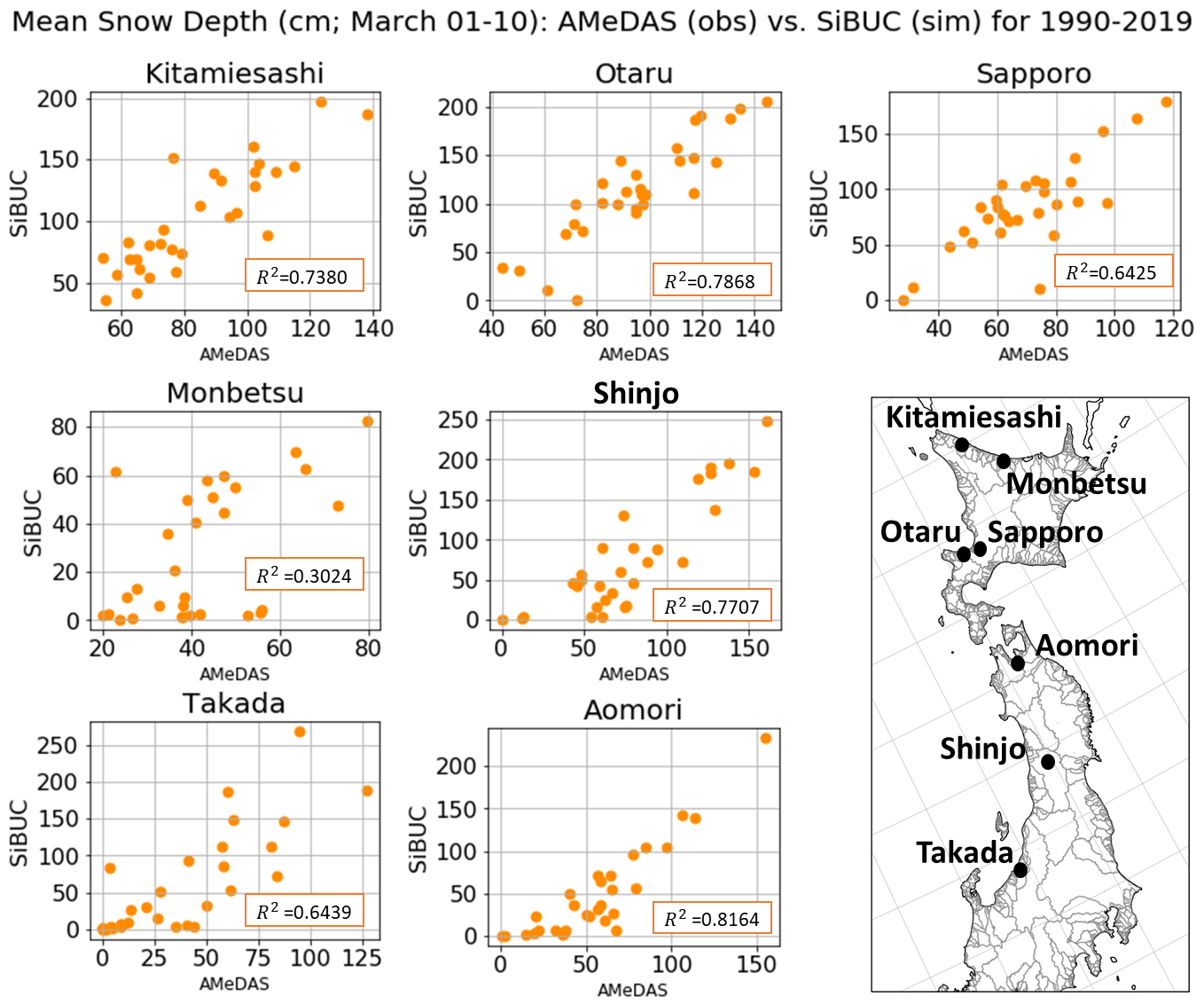


**Supplementary Figure S3.** Mean snow depth (cm) from 1^st^ to 10^th^ March determined by SiBUC-based simulations (vertical) and observation (horizontal) for 1990 to 2019.
